# Sensor-assessed grasping time as a biomarker of functional impairment in rheumatoid arthritis

**DOI:** 10.1038/s41598-025-90295-7

**Published:** 2025-02-19

**Authors:** Birte Coppers, Simon Heinrich, Koray Tascilar, Uday Phutane, Arnd Kleyer, David Simon, Johanna Bräunig, Johann Penner, Martin Vossiek, Verena Schönau, Sara Bayat, Georg Schett, Sigrid Leyendecker, Anna-Maria Liphardt

**Affiliations:** 1 Department of Internal Medicine 3 - Rheumatology and Immunology, Friedrich-Alexander-Universität Erlangen-Nürnberg and Universitätsklinikum Erlangen, Ulmenweg 18, 91054 Erlangen, Germany; 2 Deutsches Zentrum Immuntherapie, Friedrich-Alexander-Universität Erlangen-Nürnberg and Universitätsklinikum Erlangen, Erlangen, Germany; 3 Institute of Applied Dynamics, Friedrich-Alexander-Universität Erlangen-Nürnberg, Erlangen, Germany; 4 Med. Klinik mit Schwerpunkt Rheumatologie und Klinische Immunologie, Charité - Universitätsmedizin Berlin, Berlin, Germany; 5 Institute of Microwaves and Photonics, Friedrich-Alexander-Universität Erlangen- Nürnberg, Erlangen, Germany

**Keywords:** Biomarkers, Biomechanics, Fine-motor skills, Hand, Rheumatoid arthritis, Musculoskeletal system, Rheumatic diseases, Diagnostic markers, Outcomes research

## Abstract

Quantifying physical impairment in rheumatoid arthritis (RA) is important to determine disease burden and disability. Therefore, we aimed to define hand function impairments in RA patients using an opto-electronic measurement system (OMS). In this cross-sectional pilot study, spatio-temporal and hand segment data were collected during the fine motor skill Moberg Picking-Up Test (MPUT) and two elementary hand movement tasks in RA patients and healthy controls (HC) using a marker-based OMS. The duration of two MPUT movement phases (grasping, transporting 12 objects) and kinematic characteristics during the elementary movements were evaluated. We analyzed between-group differences using linear mixed-effects models accounting for within-participant clustering of hands and adjusting for age and sex. Measurements were obtained from 24 RA and 23 HC. The mean duration of the grasping phase of MPUT was longer in RA patients compared to HC while transporting times were identical, showing a significant group-phase interaction (*p* < 0.001). Interphalangeal joint angle ratios were similar in RA and HC (*p* > 0.05) with a lower ratio in both groups for the task thumb-finger opposition compared to flexion of joints. In RA patients especially grasping objects was impaired, and performance time for a subset of objects may serve as a quantitative biomarker of functional impairment.

**Supplementary Information:**

The online version contains supplementary material available at 10.1038/s41598-025-90295-7.

## Introduction

Rheumatoid arthritis (RA) patients experience a substantial decline in hand function caused by inflammation of the joints, tendons and ligaments, which especially affects the small joints of the hand^1,2^. It is known that inflammation of hand joints in RA leads to a reduction in movement accuracy^3,4^, limited range of motion^5,6^, and an overall decline of functional performance^7^. During elementary hand movements and in functional tasks the range of motion in the corresponding movement planes is limited in RA patients as a result of joint damage and/or inflammation^5,6^. It is a well-known phenomenon that the accuracy of a movement is determined by its speed, leading to lower spatial precision for faster movements. This so called “speed–accuracy trade-off” has been investigated in various models^8,9^ and is especially important during complex coordinative motor tasks. Complex motor tasks require a subtle interaction involving components such as muscle activation, proprioception and precision and might therefore be particularly prone to alterations in RA patients.

In clinical routine, hand function is rarely assessed directly but mainly based on generalized patient-reported outcome measures of physical function^10–12^. However, haptic non-electronic fine motor skill tests like the Moberg Picking-Up Test (MPUT) have been developed and validated for the use in RA patients^13^. Another important aspect of hand function is grip strength^14^. Studies have shown a decrease in strength over a five-year period, even in patients in clinical remission^7^. However, limitations exist in terms of sensitivity and feasibility. Severely impaired patients may not be able to achieve the minimum force required by a hand dynamometer, and small changes might not be captured^14,15^. While these tests are capable of detecting performance restrictions in RA compared to healthy individuals^16^, it remains unclear whether these complex functional tests can assist in continuous monitoring of function, especially with respect to changes in disease activity and early detection of flares.

In order to evaluate the importance of functional outcome measures for disease monitoring, disease-specific, direct, and quantifiable biomarkers of hand function must first be identified. In recent years, there has been growing interest in developing easily applicable tools for the assessment and quantification of hand function impairments^17–20^. While glove-based systems are often used in kinematic studies to detect functional restrictions in arthritis patients^21,22^, they may not be practical for hands of varying sizes and often lack the clinically needed accuracy^23^. Therefore, marker-less systems such as RGB-D cameras^24^ or radar-based systems^25^ are of great interest. These systems do not require sensors on the hand’s surface, allowing for unrestricted movements. Although these new sensor technologies are promising, they currently lack precision in detecting dynamic movements and still need further validation for clinical use^26^. Marker-based optoelectronic measurement systems (OMS), the current gold-standard of motion analysis, have been highly successful in assessing full body movements and may be helpful to characterize and quantify hand movement impairments^27–29^ in RA patients. To date the focus of hand movement analysis was often on the function of individual joints or conducted with methods lacking accuracy. Analyzing the interaction of finger joint kinematics during complex movements and finger interactions may provide additional information for detecting movement impairments.

We hypothesized that RA patients show impaired hand movements during fine motor skill and elementary hand movement tasks compared to healthy controls (HC). This study aimed to answer the following question: whether the use of a marker-based OMS can reveal patterns of hand movement that are characteristic for patients with RA compared to HC and provide more information than conventional hand function testing.

## Methods

### Study participants

This study protocol was approved by the Friedrich-Alexander-Universität (FAU) Erlangen-Nürnberg ethics committee (#125_16B)^20^ and all experiments were performed in accordance with the relevant guidelines and regulations. After giving written informed consent, HC (recruited over social networks of the city Erlangen, Germany) and RA patients (ACR/EULAR 2010 classification criteria^30^, recruited from the outpatient clinics of the Department of Internal Medicine 3 – Rheumatology and Immunology, Universitätsklinikum Erlangen, Germany) were included in the study. Exclusion criteria were fractures of the hand or finger bones (< 5 years) and advanced destructive alterations of the hand and finger joints. Characteristics of the participants (age, sex, anthropometrics) and handedness were recorded. RA patients had a routine clinical visit on the day of the hand function assessment. Disease duration, tender/swollen joint count (TJC/SJC 78/76), erythrocyte sedimentation rate (ESR), C-reactive protein (CRP) level, the visual analogue scale (VAS) for global disease activity, duration of morning stiffness, disease activity score 28 (DAS28)^31^ and Health Assessment Questionnaire Disability Index (HAQ-DI)^10^ were retrieved from clinical documentation.

### Conventional hand function tests and questionnaires

**Fig. 1:**
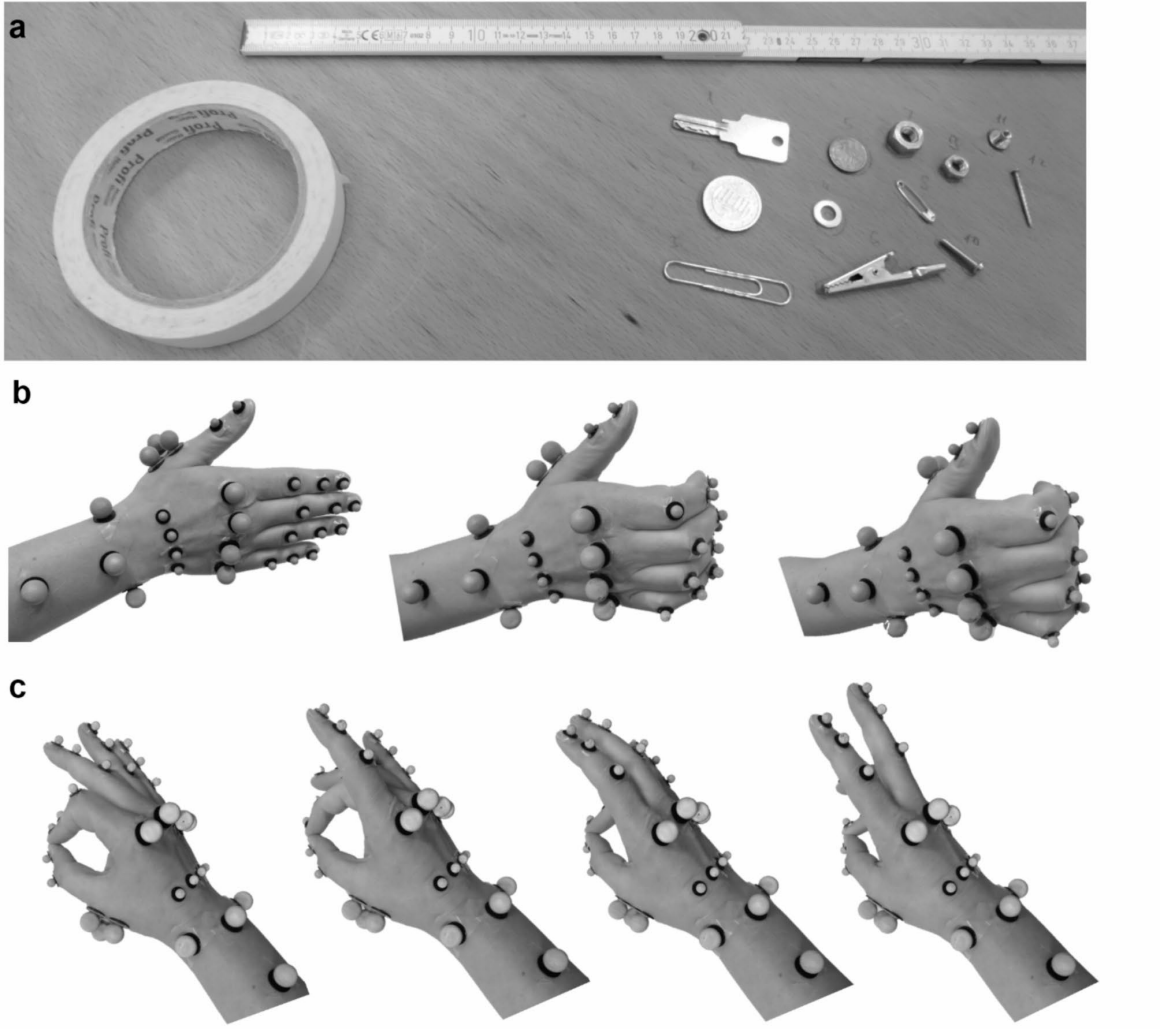
(**a**) Setup of the Moberg Picking-Up Test. Elementary hand movements: (**b**) Flexion DIP/PIP joints. (**c**) Thumb-finger opposition from index to little finger with 29 marker hand-setup.

All participants completed the Michigan Hand Questionnaire (MHQ)^32^, the shortened version of Disability of the Arm, Shoulder and Hand Questionnaire (Quick-DASH)^33^ and the Score for the Assessment and quantification of Chronic Rheumatic Affections of the Hands (SF-SACRAH)^12^. Standardized assessment of grip strength was performed using a hand dynamometer (Lafayette, IN, USA), with the highest attempts out of three trials for each hand used for analysis. Fine motor skills were tested using the MPUT: participants were asked to pick up and transport 12 small objects into a box in self-selected order at the fastest possible speed. Through marks on the table, the exact positions of the objects and the box were predetermined, ensuring identical distances between the objects and the box for each trial and participant (Fig. 1a). The task was performed twice with each hand starting with the dominant side, and the fastest of the two attempts^13^ was used for analysis.

### OMS-captured hand function

The OMS-captured hand function measurements were performed with either only one or both hands of the participants. If only one hand could be measured, data was collected from the most severely affected hand of the RA patient and the dominant hand of the HC. The MPUT was performed in the OMS setup for a second time, following the same procedure described above. Furthermore, two elementary hand and finger movements were recorded over three repetitions: *Flexion interphalangeal joints*: start in a neutral hand position with all fingers in full extension and alternating flexion and extension of the distal interphalangeal (DIP) and proximal interphalangeal (PIP) joints (Fig. 1b). *Thumb-finger opposition*: successive tapping with each finger on the tip of the thumb, starting with the index finger at self-selected speed (Fig. 1c).

### OMS analysis

**Fig. 2:**
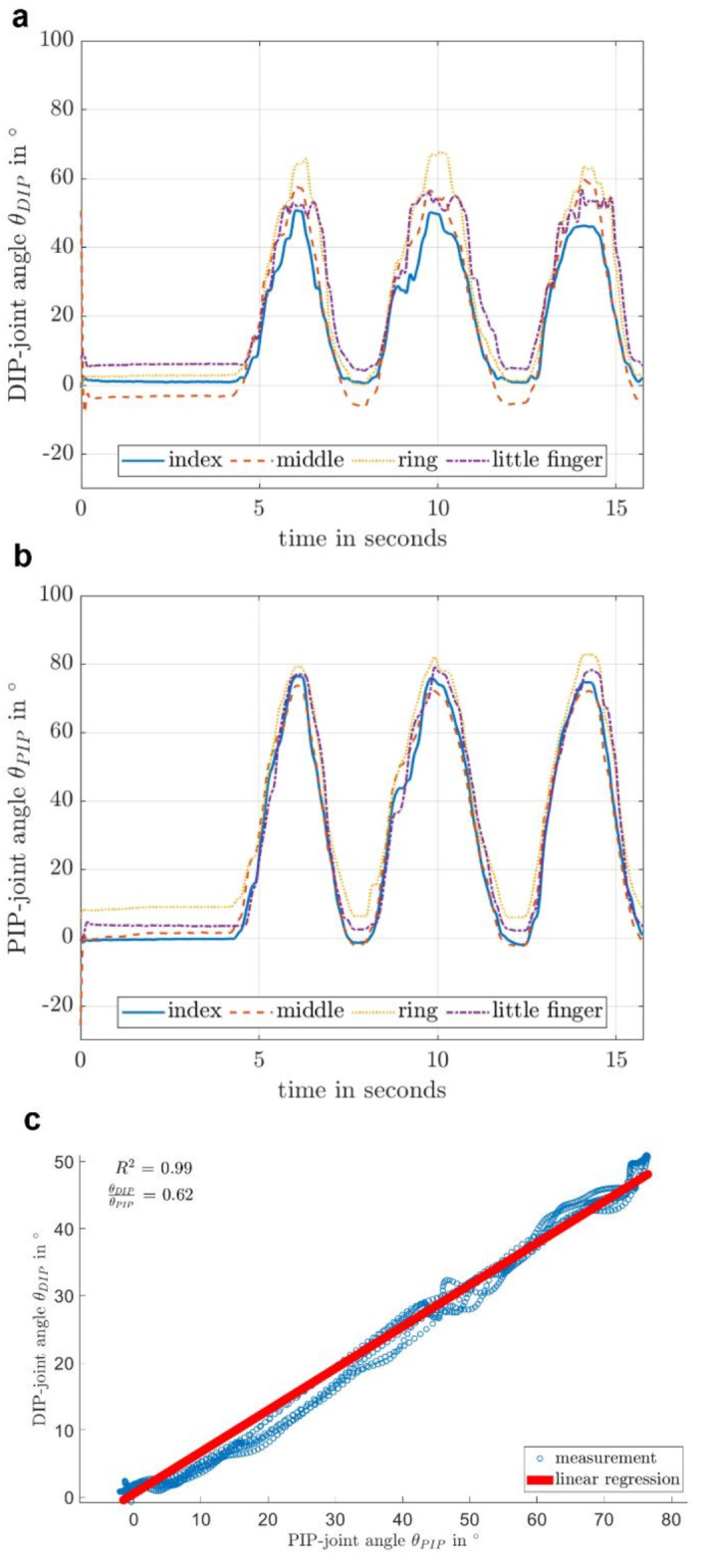
Exemplary data of one rheumatoid arthritis patient for the task flexion interphalangeal joints. (**a**) Absolute distal interphalangeal (DIP) joint flexion angle (degree positive for flexion) for all four fingers. (**b**) Absolute proximal interphalangeal (PIP) joint flexion angle for all fingers. (**c**) Measured values (blue dots) for the ratio between DIP/PIP joint angles (0.62) and linear regression (in red) for the calculation of r-squared (R^2^) (0.99) for the index finger.

A standardized 3D motion analysis was performed for the biomechanical analysis of the hand during the movement tasks using nine high-speed infrared cameras (eight Oqus7+, one Oqus5 + camera, Qualisys AB, Sweden, 100 Hz) and a standard webcam (13 Hz). 29 retroreflective markers were attached following a previously described protocol placed on bony landmarks on the hand`s dorsum, only adapted at the thumb as seen in Fig. 1^34^. Data was processed with the Qualisys Track Manager (QTM) (https://www.qualisys.com/software/qualisys -track-manager/) and MATLAB (The MathWorksInc, Version R2022a. Natick, USA). Automatic identification of markers was applied and manually corrected if necessary. Marker trajectories were low-pass filtered (Butterworth, cut-off frequency 6 Hz, 4th order) and data frames with missing markers excluded. Body-fixed coordinate systems were defined according to^34^ and in accordance with the recommendations of the International Society of Biomechanics^35^ with the x-axes positive in palmar, the y-axes in proximal and the z-axes in radial direction^34^. Anatomical angles were obtained using Z (flexion-extension)- X (adduction-abduction)- Y (pronation-supination)- Euler-angles based on the rotation matrices between neighboring segments at each time step. The OMS-based MPUT analysis focused on different movement phases, which were previously suggested for a sub-analysis of the test^20^. Three events were manually set for each object using the video recording and marker trajectories: (i) first touch for each of the 12 test objects, (ii) secure grasp of the objects, and (iii) drop when the objects fell into the box. Phases were defined as follows: grasping phase: start = event (i), end = event (ii); transporting phase: start = event (ii), end = event (iii). The DIP and PIP joint angles were analyzed for the index, middle, ring and little finger during the elementary movement tasks (Fig. 2a,b). To estimate the relationship between the dependent variable DIP and the independent variable PIP a linear model was used to compute the slope b, which represents the DIP/PIP joint angle ratio and a coefficient constant C for DIP = C + b ⋅ PIP (Fig. 2c). The coefficient of determination (R^2^) was analyzed to quantify the fit of the linear model to the obtained DIP and PIP angle values (Fig. 2c).

### Statistical analysis

The statistical analysis was performed using R (version 4.2.2, R Foundation for Statistical Computing, Vienna, Austria). Demographic, clinical, serology data and hand function questionnaires are reported as mean ± standard deviation (SD), median (inter quartile range, IQR) or percentage (n) of participants by group, based on data type and assessment of normal distribution using the Shapiro-Wilk test. Independent sample t-tests were used to assess differences in normally distributed variables and Wilcoxon Rank Sum tests for non-normally distributed variables. Significance was predetermined at *p* ≤ 0.05. For the analysis of the OMS obtained parameters with an imbalance in the number of hands per participant, within participants clustering of hands was controlled for in all statistical analysis.

To analyze group differences of the MPUT performance in each of the two settings (conventional setup and OMS) we used mixed effects linear regression models with log-transformed MPUT time values as dependent variable, age, sex and group as covariates and random intercept terms for hand dominance and participant identifier.

The same model with additional random intercept terms for object and phase and the interaction term for group-phase was used to explore whether the different movement phases during the MPUT performance in the OMS setup are affected by RA. Estimated marginal mean (EMM) and 95% confidence interval (CI) for grasping and transporting times by group were calculated. Additionally, the interaction term group-phase-object was added to the model to identify objects during a specific movement phase of the MPUT that are affected by RA. EMM for each object in the grasping and transporting phases by group were calculated and presented after ranking by absolute between-group differences.

To estimate the association between groups for the DIP/PIP joint angle ratio, and the R^2^ during the elementary movement tasks, a similar model accounting for within participants clustering of hands and fingers, adjusted for age and sex, was applied. EMM and 95% CI for DIP/PIP joint angle ratios and R^2^ values averaged over the index, middle, ring and small finger for both movement tasks were reported. OMS data was excluded from the analysis if markers were tracked poorly. Complete case analysis for the linear regression models were performed as data were assumed to be missing at random.

## Results

### Study population

24 RA patients and 23 HC were included in the study. Subject characteristics are reported in Table 1. The groups were balanced in terms of sex and anthropometrics, yet the RA patients were significantly older than HC (*p* = 0.02). No group differences were detected for number of smokers and alcohol consumption, but a greater number of HC compared to RA patients participated in regular sporting activities (> 2 per week, *p* < 0.01). Disease activity in RA patients was low (DAS28 = 2.6 ± 1.3, mean ± SD) and patients rated themselves mostly in the lower third of the VAS range with respect to global disease activity (median 18.0, IQR 7.8 to 36.0).

**Table 1:** Characteristics of rheumatoid arthritis patients and healthy controls.

	Rheumatoid arthritis (*n* = 24)	Healthy controls (*n* = 23)	*p*- value
Demographics			
Sex, % female (no.)	70.8 (17)	50.2 (12)	0.19
Age, years, mean ± SD	62.3 ± 9.1	50.2 ± 16.1	0.02
BMI, kg/m2, median (IQR)	25.1 (8.0)	26.1 (6.3)	0.7
Alcohol intake, % (no.)	70.8 (17)	69.6 (16)	0.92
Smoking, % (no.)	37.5 (9)	17.4 (4)	0.12
Regular sports activities, % (no.)	9.5 (2)	55.4 (12)	< 0.01
Serology			
Rheumatoid factor, IU/ml, median (IQR)	52.64 (516.53)		
Anti-MCV, U/ml, median (IQR)	10.05 (110.93)		
Anti-CCP, RU/ml, median (IQR)	5.11 (119.32)		
ESR, mm/hour, mean ± SD	16.1 ± 10.2		
CRP, mg/l, mean ± SD	14.2 ± 4.8		
Vitamin D, ng/ml, mean ± SD	35.2 ± 8.8		
Clinical scores			
TJC/SJC 66/68, no., median (IQR)	0 (3.25)/ 0 (1.25)		
DAS28 CRP, 0–10 units, mean ± SD	2.6 ± 1.3		
HAQ-DI, 0–3 units, median (IQR)	0.25 (0.94)	0.00 (0.06)	< 0.001
VAS global, 0-100 units, median (IQR)	18.0 (28.25)		
Morning stiffness, min, median (IQR)	5 (10)		
Disease duration, years, median (IQR)	8.1 (12.8)		
Ulnar deviation present, % (no.)	4.8 (1)	0.0 (0)	0.30
Dactylitis present, % (no.)	4.3 (1)	0.0 (0)	0.31
Hand function			
MHQ total, 0-100 units (%), median (IQR)	69.2 (23.8)	93.9 (4.9)	< 0.001
MHQ ADL, 0-100 units (%), median (IQR)	93.0 (16.2)	100.0 (0.9)	< 0.001
Quick-DASH, 0-100 units, median (IQR)	22.7 (18.2)	2.3 (6.0)	< 0.001
SF-SACRAH, 0–10 units, median (IQR)	0.0 (0.0)	0.6 (1.65)	< 0.001
Grip strength, pounds, mean ± SD	71.5 ± 29.6	91.7 ± 35.7	0.02

*SD* standard deviation, *BMI* body-mass-index, *ESR* erythrocyte sedimentation rate, *CRP* C-reactive protein, *Anti-MCV* Anti mutated citrullinated vimentin, *CCP* cyclic citrullinated peptide, *TJC/SJC* tender joint count/swollen joint count, *VAS global* visual analogue scale global disease activity, *DAS28* disease-activity-score − 28, *HAQ-DI* health assessment questionnaire disability index, *MHQ total* Michigan hand questionnaire total score, *ADL* activities of daily living, *Quick-DASH* disability of the arm, shoulder and hand questionnaire, *SF-SACRAH* short form of the assessment and quantification of chronic rheumatic affections of the hands.

### Conventional hand function tests and questionnaires

Patient-reported outcome measures and hand function tests indicated a higher level of functional impairment (HAQ-DI), perceived hand impairment (MHQ, Quick-DASH, SF-SACRAH) (*p* < 0.001) (Table 1) and reduced grip strength (*p* = 0.017) (Table 1) in RA patients compared to HC. Age and sex adjusted measured performance time of the MPUT in the conventional setup was increased in RA patients compared to HC (+ 14%, 95% CI, −1% to + 30%), with EMM of 16.05 s for RA and 14.10 s for HC.

### OMS captured hand function

OMS-based motion analysis was performed with 29 hands of the 24 RA patients and 35 hands of the 23 HC.

#### Moberg picking-up test

For the OMS-based MPUT analysis, measurements of all assessed hands could be included. The same between-group difference for MPUT times in the conventional setup was observed in the OMS setup with slightly increased uncertainty (+ 14%, 95% CI −3% to + 35%) with EMM of 18.38 s for RA and 16.07 s for HC. The overall OMS-MPUT times in the OMS set-up were affected by age (+ 10% per 10 years, 95% CI + 4% to + 17%).

**Fig. 3:**
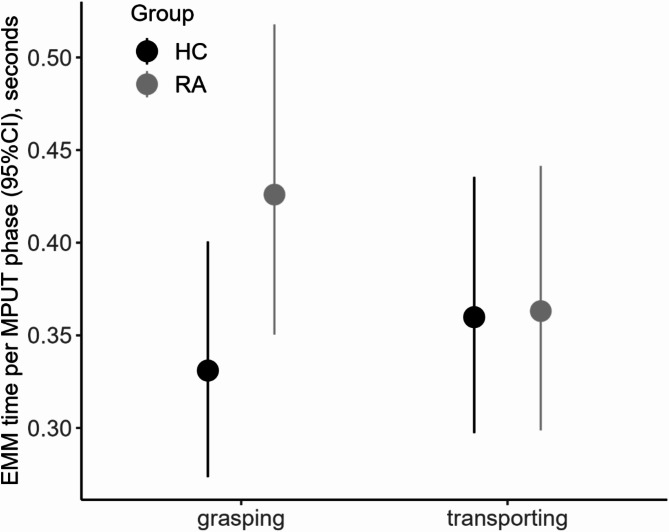
Estimated marginal means (EMM) for the group-phase interaction model. Grasping and transporting times for the mean overall age and averaged over males and females by group.

For the MPUT performance time measured in the OMS setup, the interaction model revealed a significant group-phase interaction (*p* < 0.001). EMM (95%CI) for the grasping phase times across all objects showed higher absolute differences between the groups (RA 0.43 (0.35 to 0.52) s, HC 0.33 (0.27 to 0.40) s), while transporting times were identical for RA patients (0.36 (0.30 to 0.44) s) and HC (0.36 (0.30 to 0.44) s) (Fig. 3). RA patients on average took longer for grasping the objects, while HC tended to spend more time for transporting them to the box compared to grasping the objects (Fig. 3). MPUT movement phase was affected by age (*p* < 0.001), whereas no sex-related differences were observed (*p* = 0.46).

**Fig. 4:**
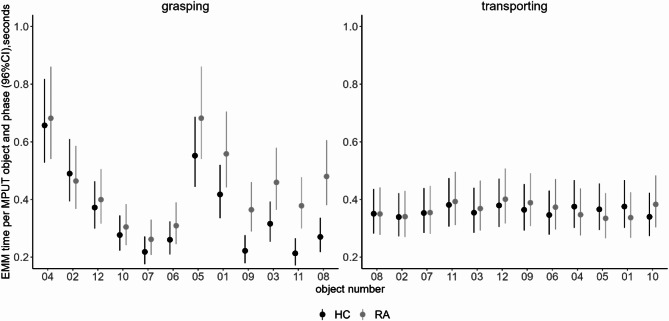
Age and sex adjusted estimated marginal means (EMM) for MPUT times per object and group divided by grasping and transporting phase from group-phase-object interaction model ranked by absolute between-group differences. Object 1 = key, 2 = 50-Cent coin, 3 = paper clip, 4 = shim, 5 = 1-Cent coin, 6 = clip, 7 = screw nut 1.4 cm, 8 = safety pin, 9 = screw nut 1.0 cm, 10 = screw 2.3 cm, 11 = threaded sleeve, 12 = thin nail.

The interaction model (group-object-phase) revealed that in the MPUT grasping phase specific MPUT objects are indicative for group differences. Unadjusted means, 95% CI and unadjusted absolute mean differences of sensor measured grasping and transporting times by object and group are presented in Supplementary Table 1. Calculated EMM for the time of MPUT grasping phase of object (8) *safety pin* and object (11) *threaded sleeve* showed slower performance times in RA patients compared to HC and the highest absolute time difference between the groups (0.21, 0.17 s respectively) (Fig. 4). Also, the objects (3) *paper clip*, (9) *screw nut*, (1) *key*, (5) *1-Cent coin* showed relatively high between-group differences compared to the other six objects (0.14, 0.14, 0.14, 0.13 s respectively). For all objects, except object (2) *50-Cent coin*, the model revealed slower grasping phase times for RA compared to HC (Fig. 4).

#### Elementary hand movements

Descriptive statistics (mean ± SD) of the unadjusted data for the determined slope (DIP/PIP joint angle ratio), constant and R^2^ in the regressions across all participants per finger, group and movement task are presented in Table 2. The EMM (95% CI) derived from the linear model as averaged over all fingers and at the mean overall age, average for males and females are presented in Table 3.

**Table 2:** Unadjusted mean and standard deviation of the slope (DIP/PIP joint angle ratio), constant (in degree) and r-squared values in the regression for each finger during the two movement tasks by group.

Elementary movements	Rheumatoid arthritis			Healthy controls		
Mean ± SD	Slope	Constant	R^2^	Slope	Constant	R^2^
Interphalangeal joint flexion						
Index (II)	0.56 ± 0.12	−1.28 ± 7.32	0.95 ± 0.06	0.62 ± 0.17	-1.54 ± 6.31	0.96 ± 0.04
Middle (III)	0.62 ± 0.13	−2.66 ± 5.43	0.96 ± 0.04	0.71 ± 0.17	-2.36 ± 7.37	0.97 ± 0.02
Ring (IV)	0.58 ± 0.16	−2.32 ± 8.51	0.96 ± 0.05	0.68 ± 0.14	-3.41 ± 6.57	0.96 ± 0.03
Little (V)	0.65 ± 0.17	−3.13 ± 9.12	0.96 ± 0.04	0.73 ± 0.17	-0.96 ± 7.72	0.97 ± 0.02
Thumb-finger opposition						
Index (II)	0.52 ± 0.19	1.71 ± 7.19	0.75 ± 0.22	0.50 ± 0.18	-3.02 ± 6.10	0.76 ± 0.19
Middle (II)	0.52 ± 0.16	−0.48 ± 5.61	0.76 ± 0.18	0.54 ± 0.14	-2.37 ± 6.40	0.82 ± 0.17
Ring (IV)	0.47 ± 0.21	0.21 ± 9.39	0.66 ± 0.23	0.48 ± 0.19	-1.02 ± 6.60	0.71 ± 0.19
Little (V)	0.54 ± 0.36	−0.67 ± 0.65	0.63 ± 0.35	0.60 ± 0.20	0.69 ± 6.91	0.81 ± 0.19

*SD* standard deviation, *DIP* distal interphalangeal, *PIP* proximal interphalangeal.

**Table 3:** Estimated marginal mean (EMM), 95% confidence interval (CI) for the kinematic measures of DIP/PIP ratio and r-squared (R^2^) averaged over all fingers and hands during the two movement tasks by group.

EMM (95% CI)	Rheumatoid arthritis	Healthy controls	*p*-value
Interphalangeal joint flexion			
DIP/PIP joint angle ratio	0.62, 0.53 to 0.71	0.65, 0.56 to 0.74	0.1
R^2^	0.96, 0.95 to 0.97	0.97, 0.96 to 0.98	0.3
Thumb-finger opposition			
DIP/PIP joint angle ratio	0.54, 0.46 to 0.61	0.52, 0.45 to 0.60	0.79
R^2^	0.72, 0.65 to 0.79	0.77, 0.70 to 0.84	0.23

*DIP* distal interphalangeal, *PIP* proximal interphalangeal.

For the task *flexion interphalangeal joints* two hands of HC and one hand of an RA patient needed to be excluded from analysis due to failed marker tracking. This resulted in the analysis of 33 hands of 22 HC and 28 hands of 24 RA patients for this movement task. The mean of the unadjusted data for the parameter DIP/PIP joint angle ratio ranged between 0.56 and 0.65 across all fingers for RA patients and between 0.62 and 0.73 for HC (Table 2). The calculated EMM for the DIP/PIP joint angle ratio during task *flexion interphalangeal joints* revealed a slightly lower DIP/PIP joint angle ratio in RA patients compared to HC but no statistical differences between the groups was identified for this parameter (Table 3). The mean of R^2^ for the unadjusted data ranged between 0.95 and 0.96 for RA patients and 0.96 to 0.97 for HC (Table 2). The calculated EMM for R^2^ revealed similar results and no differences between the groups could be identified (Table 3).

For the analysis of the task *thumb-finger opposition*, three hands of HC and two hands of RA patients needed to be excluded from analysis due to failed marker tracking. This resulted in 32 hands of 23 HC and 27 hands of 22 RA patients in this analysis. DIP/PIP joint angle ratio over the whole movement performance of *thumb-finger opposition* showed lower ratios compare to the task *flexion interphalangeal joints*. On average the ratio ranged between 0.48 and 0.60 for RA patients and 0.47 to 0.54 for HC for unadjusted data (Table 2). Also, the averaged DIP/PIP joint angle ratios showed similar results and the statistical analysis revealed no differences between the groups (Table 3). Overall, R^2^ was lower for the task *thumb-finger opposition* compare to the task *flexion interphalangeal joints*. The mean of the unadjusted data ranged between 0.63 and 0.75 within RA patients and 0.71 to 0.82 for HC. No differences between the groups could be identified, while the group of RA patients presented with a slightly lower fit compared to HC (Table 3).

## Discussion

This pilot study aimed to identify and quantify patterns of hand movement impairments in RA patients analyzing a complex fine motor task (MPUT) and elementary hand movements using a marker-based OMS.

MPUT performance in the OMS set-up is feasible and provides reliable results as RA patients and HC experienced a similar decrease in movement speed due to the markers on the hand palm. Sub-phase analysis of fine motor task performance revealed disease-related functional impairments in the grasping phase in RA patients compared to healthy subjects while transporting objects was similar in both groups. In particular the grasping time for flat and small objects like the safety pin and threaded sleeve, respectively, demonstrated the highest between-group differences. These findings suggest that in RA patients specific hand functions are more prone for impairment by the disease, as previously proposed by^20^. These data reflect findings in gait analysis tests like the Timed-up-and-Go Test, where the specific functions like sitting, walking and turning, yield more information on patient’s mobility than the overall completion time^36,37^. Also, analyzing kinematic synergies in hand function tests like the Sollerman Hand Function Test identified specific tasks that are more responsive to detecting hand osteoarthritis^38^.

The conventional MPUT is well-suited for assessing functional impairments^16^; the test set-up is simple and not time consuming (set up and completion can be achieved in five minutes). For a more detailed monitoring of disease activity and treatment response, our findings suggest the potential for adapting the test. By using specific objects and measuring grasping time, the test could offer a more sensitive assessment, providing a direct and quantitative biomarker of hand function, which needs to be investigated in further studies.

Furthermore, our analysis confirms that combined assessment of speed and spatial accuracy can reveal functional limitations affecting the motor performance^3,39^. Especially the realization of a precision grasp under time pressure appears to be difficult for RA patients, and the unskillfulness of manipulating small objects caused by disease-related impairment of hand function is reflected in longer duration of the grasping phase. Usually reduced motor skills in RA patient are explained by active inflammation leading to pain and swelling^30^. However, as RA patients participating in this study were in remission or had low disease activity, acute inflammatory symptoms may not completely explain impaired function. A possible explanation is that inflammation leads to structural changes of tendons, ligaments, cartilage and bone, which may influence complex hand function^40–42^. Next to structural lesions changed adaptations in the neuromuscular system may be responsible for this sustained alteration in hand function^43^. Of note, grasping objects requires the precise coordination of concentric work by the flexor muscles of the fingers and thumb, while simultaneously controlling the extension of the extensor muscles and tendons. Altered movement strategies that arose during phases of high disease activity appear to remain fixed in phases of low disease activity thereafter. To deepen the understanding of the impact of structural lesions and neuromuscular alterations in participating in fixed function impairment in RA patients, tools like ultrasonography^44^ and electromyography may be needed^45,46^. Future studies could focus on assessing muscle activity patterns during functional hand movement tasks with respect to underlying physiological alterations.

Biomechanical endpoints have not been analyzed during the performance of the elementary movements *flexion interphalangeal joints* and *thumb-finger opposition* in RA patients previously. Essentially the kinematic components analyzed during these elementary movements were not different in RA patients and HC confirming the concept that more complex functional task rather than elementary tasks are primarily affected in RA. Hence, our data show a linear relationship between the DIP and PIP joint angles during free movements at an angle ratio of approximal 2/3 in RA and almost 100% of the variability in DIP joint angle values explained by the linear regression model, which is similar to HC^47,48^. During the task *thumb-finger opposition*, the interaction with the thumb`s surface influenced the joint angle ratio, resulting in a lower DIP/PIP angle ratio and linear fit of the measured data. This was observed in both groups, which is consistent with previous findings in HC^48^. While elementary movements were not altered in RA patients, measurements such as DIP/PIP flexion might be helpful in the assessment of patients with psoriatic arthritis^49^ or osteoarthritis, where DIP joints are more frequently affected by the disease^38^.

Integration of an OMS setup into the clinical routine is clearly not practical and therefore served solely the purpose to precisely quantify the hand function impairments. In the future, applications such as non-contact sensor systems, e.g. using RGB-D cameras^24,26^ will be needed to provide a patient-centered tool for monitoring functional impairment.

While we aimed at reaching a high level of standardization with our study protocol, this study has some limitations. Due to the complexity of the study protocol and the pilot-study nature of this trial, the sample size is small and age was imbalanced between the groups. Statistical analysis was adjusted for age and sex differences. Speed and strength of movement are known to be influenced by these factors^7,16,50^. Therefore, it will be essential for future studies to include a broader age range in study groups to establish valid reference values for different age and sex groups. Another limitation is the restricted range of disease activity in the included RA patients. A wider range of disease activity would be desirable to get a clearer understanding of movement impairment associated with disease activity. On the other hand, functional impairment during active arthritis is obvious whereas the distinct functional impairments accumulating in RA patients in remission easily escape detection but are relevant for the long-term outcome of these patients.

An important challenge in hand motion analysis is the high variability of movement performance. Unlike highly standardized gait analysis, which relies on defined events such as foot contact with the ground and the cyclic nature of walking, standardized assessment of hand motion remains rare^29,51^. Standardized assessment proved crucial during the analysis of the *flexion interphalangeal joints* task. Unclear instructions and individual variation in performance led to incomplete joint flexion and extension, necessitating the exclusion of range of motion analysis. This observation highlights the necessity of carefully selecting biomechanical endpoints in line with clear movement instructions.

Fine motor skills and grip strength depend on individual experience and familiarity with the task. RA patients and HC both took the longest time for grasping a small coin, and no difference between the groups was observed. Likely due to the inherent difficulty of the task, there was a high inter-subject variation within both groups resulting in no disease-specific difference detected for grasping this object. Thus, differences between persons might occur due to inter-personal characteristics independently of the factor “RA diagnosis” and within persons as an effect of the individual experience with this task. Therefore, further studies should focus on identifying specific tasks that are related to the target disease and disease activity and allow to individually monitor hand function in prospective long-term studies.

In conclusion, our data imply that RA patients present with disease-related impairments in the task of grasping compared to transporting objects. Furthermore, we observed that a certain subset of the objects contributed more in distinguishing RA patients from HC. In contrast to complex tasks, in which the functional impairment of RA patients is unmasked, some kinematic components during elementary movements remain obviously stable in the majority of RA patients. Hence, the assessment of hand function focusing on competencies like speed and spatial accuracy, even in patient with clinical low disease activity, reveals additional information on functional limitations compared to conventional hand function tests. Therefore, in-depth analysis of hand movements contributes to a better understanding of functional impairment and holds the potential to serve as a further component for disease monitoring even among patients in remission or with low disease activity.

## Electronic supplementary material

Below is the link to the electronic supplementary material.

Supplementary Material 1

## Data Availability

The datasets generated and analyzed during the current study are available from the corresponding author on reasonable request.
