# Supplementary material for: Sensor-assessed grasping time as a biomarker of functional impairment in rheumatoid arthritis
Source: Sci Rep. 2025 Feb 19;15:6018. doi: 10.1038/s41598-025-90295-7 (PMC11840059; doi:10.1038/s41598-025-90295-7)
Supplement: Supplementary file 1 — Supplementary Material 1 [file 41598_2025_90295_MOESM1_ESM.docx]

Supplementary Table 1. Unadjusted means, 95 % confidence intervals and unadjusted absolute mean differences (seconds) of sensor measured grasping and transporting times by object and group, ranked by absolute between-group mean difference.

| Phase | Object | Mean difference | Healthy Controls | Rheumatoid Arthritis |
| --- | --- | --- | --- | --- |
| grasping | 08 | 0.410 | 0.29 (0.24 to 0.34) | 0.70 (0.47 to 1.04) |
| grasping | 01 | 0.362 | 0.48 (0.37 to 0.62) | 0.84 (0.51 to 1.37) |
| grasping | 03 | 0.338 | 0.38 (0.27 to 0.52) | 0.71 (0.47 to 1.00) |
| grasping | 11 | 0.278 | 0.25 (0.20 to 0.31) | 0.53 (0.38 to 0.70) |
| grasping | 05 | 0.253 | 0.68 (0.49 to 0.93) | 0.93 (0.66 to 1.30) |
| grasping | 09 | 0.217 | 0.25 (0.20 to 0.32) | 0.47 (0.36 to 0.60) |
| grasping | 02 | 0.183 | 0.59 (0.44 to 0.75) | 0.77 (0.41 to 1.37) |
| grasping | 12 | 0.165 | 0.62 (0.33 to 1.03) | 0.78 (0.36 to 1.36) |
| grasping | 04 | 0.094 | 0.88 (0.61 to 1.20) | 0.98 (0.70 to 1.28) |
| grasping | 10 | 0.090 | 0.41 (0.28 to 0.57) | 0.50 (0.29 to 0.83) |
| transporting | 10 | 0.081 | 0.33 (0.30 to 0.36) | 0.41 (0.37 to 0.46) |
| transporting | 06 | 0.078 | 0.34 (0.31 to 0.36) | 0.41 (0.35 to 0.48) |
| transporting | 09 | 0.075 | 0.35 (0.33 to 0.37) | 0.42 (0.37 to 0.49) |
| grasping | 06 | 0.072 | 0.34 (0.24 to 0.46) | 0.41 (0.31 to 0.53) |
| transporting | 03 | 0.053 | 0.34 (0.32 to 0.37) | 0.40 (0.36 to 0.44) |
| transporting | 11 | 0.047 | 0.37 (0.34 to 0.41) | 0.42 (0.38 to 0.47) |
| grasping | 07 | 0.044 | 0.27 (0.19 to 0.35) | 0.31 (0.25 to 0.38) |
| transporting | 12 | 0.042 | 0.37 (0.34 to 0.41) | 0.41 (0.36 to 0.46) |
| transporting | 02 | 0.041 | 0.33 (0.30 to 0.35) | 0.37 (0.33 to 0.41) |
| transporting | 07 | 0.035 | 0.34 (0.32 to 0.36) | 0.37 (0.34 to 0.41) |
| transporting | 08 | 0.033 | 0.34 (0.31 to 0.37) | 0.37 (0.34 to 0.42) |
| transporting | 04 | 0.007 | 0.38 (0.33 to 0.47) | 0.37 (0.33 to 0.42) |
| transporting | 01 | 0.005 | 0.36 (0.34 to 0.39) | 0.37 (0.32 to 0.43) |
| transporting | 05 | 0.005 | 0.36 (0.33 to 0.39) | 0.36 (0.32 to 0.40) |
| Object 1 = key, 2 = 50-Cent coin, 3 = paper clip, 4 = shim, 5 = 1-Cent coin, 6 = clip, 7 = screw nut 1.4cm, 8 = safety pin, 9 = screw nut 1.0cm, 10 = screw 2.3 cm, 11 = threaded sleeve, 12 = thin nail | | | | |
